# The Hippocampus Sparing Volume Modulated Arc Therapy does not Influence Plan Quality on Locally Advanced Nasopharyngeal Carcinoma Patients

**DOI:** 10.1038/s41598-017-03517-y

**Published:** 2017-06-13

**Authors:** Wendong Gu, Qilin Li, Dan Xi, Ye Tian, Juncong Mo, Honglei Pei

**Affiliations:** 1 Department of Radiation Oncology, The Third Affiliated Hospital of Soochow University, The First Peoples’ Hospital of Changzhou, Changzhou, 213003 China; 2 Department of Radiation Oncology, The Second Affiliated Hospital of Soochow University, Suzhou, 215004 China

## Abstract

Irradiation on hippocampus would lead to neuro-cognitive dysfunction in locally advanced nasopharyneal carcinoma (LA-NPC) patients accepting radiotherapy. Our study here aimed to investigate if undergoing hippocampus sparing (HS) volume modulated arc therapy (VMAT) would influence the plan quality in LA NPC patients. We designed three kinds of radiotherapeutic plans for 11 LA NPC patients: conventional VMAT (C-VMAT), HS-VMAT and HS intensity modulated radiation therapy with dynamic multileaf collimator (HS-dMLC). And the dose distribution on targets and surrounding organs at risk (OAR) were carefully evaluated. We found out that the expected doses on hippocampus were significantly lowered in HS-VMAT (899 ± 378 cGy) and HS-dMLC (896 ± 321 cGy) as compared to C-VMAT (1518 ± 337 cGy, p < 0.05), but meaningless difference was presented on plan quality of targets (p > 0.05). Moreover, lower radiation doses on brain stem were observed in HS-VMAT plan in comparison with C-VMAT plan (p < 0.05). However, there were no statistically meaningful diversities in the doses received by other OARs among all plans. Here we concluded that HS-VMAT presented promising advantages on protecting hipppcampus and brain stem as compared to C-VMAT and HS-dMLC, but enthusiastically had no effects on plan quality in LA-NPC patients.

## Introduction

Radical radiotherapy is now considered as the first line therapeutic strategy against locally advanced nasopharyngeal carcinoma (LA NPC)^1, 2^. Yet this radiotherapeutic strategy was reported to present severe side effects of a decline in memory and social understanding after treatment^3^. And such neuro-cognitive dysfunction was later found out to be attributed to radiation damage on the hippocampus dentate gyrus caused by radiation treatment^4^. However, intensity modulated radiation therapy (IMRT)^5^, including Volumetric Modulated Arc Therapy (VMAT)^6, 7^, has been widely used in NPC patients. And therapeutic plans can help to maximize the conformal radiation dose on the targets and minimize the dose on surrounding normal organs at risk (OAR). But unfortunately no extra attention was paid to hippocampus when designing radiotherapeutic plans in NPC patients, which caused hippocampus to receive excessive radiation damage during treatment. Lowering the radiation dose on hippocampus would help reserving the neuro-cognitive capacity and improving the life quality of patients^8, 9^. Our study aimed to verify if taking hippocampus into surrounding OARs is available in LA NPC patents receiving radiological therapy (IMRT or VMAT).

## Methods and Meterials

### Patients

This was a treatment planning study and had been approved by the Institutional Review Board of the The First People’s Hospital of Changzhou, and written informed consent was obtained from the patients before treatment. The methods used in this study were carried out in accordance with the guidelines outlined in the Declaration of Helsinki. 11 LA NPC patients were recruited into our study with 6 ones at T3 stages and 5 ones at T4 stages (Supplemental Table 1). The plans were designed in the Monaco 5.10 treatment planning system (TPS, Elekta, Stockholm, Sweden) and a 6 MV photon beam (Axesse with the leaf 5mm width at the isocenter, Elekta, Stockholm, Sweden). Before performing planning design, all patients received computed tomography (CT) and magnetic resonance imaging (MRI), then all the images were transferred to Monaco 5.10 TPS for further processing.

### Targets delineation

CT and T1-weighted (T1W) MRI images were manually rigidly co-registered using the Monaco 5.10 TPS by two senior radiation oncology specialists. According to *The consensus on the target’s delineation for NPC among Chinese experts 2010* ^10^ and RTOG 0615 protocol^11^, targets and surrounding normal organs were contoured: (1) GTVnx: visible nasopharyngeal neoplasms in the images of MRI and planning CT; (2) GTVnd: cervical lymph node metastasis; the retropharyngeal lymph node metastasis was not defined separately; (3) CTV: adding 0.5 cm~1 cm margin to GTVnx and including the posterior nasal, posterior maxillary sinus, pterygopalatine fossa, part of ethmoid sinus, parapharyngeal gap, the base of the skull, part of the cervical vertebrae, the clivus and cervical lymph drainage zones; for simplifying the study, all cervical lymph drainage zones were seen as a part of CTV. (4) PTVnx and PTV were generated by expanding the GTVnx and CTV 5 mm; (5) The hippocampus were delineated on the fused images, based on the established anatomical guidelines by Chera *et al*.^12^ and the RTOG hippocampal atlas^13^.

### The Radiotherapeutic Plans and Prescriptions of radiation doses

We designed three radiotherapeutic plans of conventional VMAT (C-VMAT), hippocampus sparing VMAT (HS-VMAT), and HS intensity modulated radiation therapy with dynamic multileaf collimator (HS-dMLC) for each LA NPC patient recruited into our study. The prescription of radiation dose for C-VMAT was listed in Table 1, without taking the protection of the hippocampus into consideration. There was one dual-arc beam (from 180° to −180° then returned to 180°, maximum 200 segments in total), and the nominal dose rate was 660 MU/min. HS-VMAT was a plan different from C-VMAT as it was supposed to achieve hippocampus protection through dose-volume limit in the prescription, and V7.3 (the volume of the hippocampus received 7.3 Gy) was designated to be less than 40%. HS-dMLC was to some extent same as HS-VMAT, but the delivery mode was altered to the dynamic multileaf collimator. There were 9 equidistant, coplanar fields (the gantry angle was zero for the first beam); the maximum number of segments in one beam was set to 15.

**Table 1:** The prescription for all the plans.

Tissue Name	For Target		For Organs at Risk		
	95% volume(Rx)	Volume of 110%Rx	1% volume	Dose volume constrict	Maximum dose
PTV	60				
PTVnx	70	<2%			
GTVnd	70	<2%			
Spinal Cord			40		45
Lenses					8
Brain Stem			54		58
Chiasm					54
Optic Nerve					54
Larynx				V50% < 40 Gy	
Parotid				V50% < 35 Gy	
Middle ear					
*Hippocmapus*					
C-VMAT				N/A	
HS-VMAT				V40% < 7.3 Gy	
HS-dMLC				V40% < 7.3 Gy	

### Plan evaluation

Evaluation was processed based on the data from dose-volume histogram (DVH) for the targets (PTVnx, GTVnd and PTV). And main evaluation parameters reported by now were D98% (Dx%: the dose covered x% volume of the tissue), D50%, D2% (representing the maximum dose), Dmean (the mean dose received by tissues), conformity index (CI) and homogeneity index (HI).

In our study, we evaluated the parameters of Dmean and D40%, V10 (Vx: the percent of volume receive x Gy), V20, V30,V40, V50 on hippocampus, parameters of V10, V20, V30,V40, V50 on Brain stem and Cerebellum, D2% and Dmean on other OARs.

#### Conformity index (CI) and homogeneity index (HI)

The formulas of CI and HI^14^ were defined as follows:

1 $$HI=(D{2} \% -D{98} \% )/D{50} \% $$

2 $$CI=(T{V}_{{\rm{RI}}}\times T{V}_{{\rm{RI}}})/(TV\times {V}_{{\rm{RI}}})$$

where V_RI_ is the total volume in the body receiving the prescribed dose, TV is the target volume, and TV_RI_ is the volume of TV within the V_RI_. The ideal and maximum CI value is 1, and bigger CI indicated better conformality. The ideal and minimum HI value is 0, and smaller HI indicated better homogeneity.

#### The monitor units (MU), the number of segments and the treatment times

The MUs, the number of segments and the treatment times for each plan were recorded and compared, and they mainly implied the delivery efficiency of the plans.

### Statistics

Statistical analysis was performed on the SPSS version 18 software (IBM SPSS Statistics, NewYork, America). And data were analyzed by one-way analysis of variance (ANOVA One-Way) and the least-significant difference method (LSD), with p < 0.05 considered as statistical significance.

## Results

### HS-VMAT plan had no effect on the dose parameters for targets as compared to C-VMAT

The dosimetric parameters for the targets in all the three plans were presented in Table 2. No statistical significance was observed in the dose coverage of targets in all the 11 LA NPC patients in the two plans of C-VMAT and HS-VMAT (p > 0.05). However, HI of PTV and D_2%_ of GTVnd in HS-dMLC (0.22, 7420 cGy) seemed to be better than those in C-VMAT (0.239, 7522 cGy) and HS-VMAT (0.241, 7501 cGy), which indicated better homogeneity in HS-dMLC plan. Moreover, HS-dMLC presented better MU and numbers of segments (126, 856.7MU) as compared to C-VMAT (181, 972.3MU) and HS-VMAT (178,985.2MU), indicating that HS-dMLC would have higher delivery efficiency than the other two plans. But unfortunately HS-dMLC had longer treatment time of 426.9 s, almost two times of those in C-VMAT (189.6 s) and HS-VMAT (182.8 s).

**Table 2:** Dose coverage for the targets and the planning parameters. (Plan A: C-VMAT, Plan B: HS-VMAT, PlanC: HS-dMLC).

	Plan A	Plan B	Plan C	P value			
	mean	mean	mean		A Vs B	B Vs C	A Vs C
PTVnx							
D_98%_ (cGy)	6989	6986	6950	0.728	0.95	0.515	0.476
D_95%_ (cGy)	7053	7045	7054	0.95	0.791	0.776	0.985
D_50%_ (cGy)	7273	7260	7237	0.196	0.496	0.261	0.076
D_2%_ (cGy)	7542	7513	7462	0.016	0.289	0.058	0.005
D_mean_ (cGy)	7267	7253	7230	0.191	0.487	0.26	0.074
HI	0.076	0.073	0.071	0.827	0.703	0.822	0.545
CI^*^	0.55	0.56	0.63	0.245	0.915	0.163	0.135
PTV							
D_98%_ (cGy)	5837	5812	5914	0.029	0.513	0.011	0.049
D_95%_ (cGy)	5988	5972	6039	0.089	0.616	0.037	0.103
D_50%_ (cGy)	6526	6505	6450	0.238	0.641	0.235	0.103
D_2%_ (cGy)	7399	7380	7333	0.047	0.476	0.08	0.017
D_mean_ (cGy)	6574	6556	6529	0.435	0.602	0.446	0.203
HI	0.239	0.241	0.220	0.016	0.814	0.009	0.016
CI	0.801	0.802	0.821	0.386	0.995	0.235	0.233
GTVnd							
D98% (cGy)	6994	6994	6914	0.548	0.998	0.345	0.346
D95% (cGy)	7048	7046	7053	0.966	0.93	0.798	0.867
D50% (cGy)	7255	7240	7220	0.301	0.501	0.378	0.125
D2% (cGy)	7522	7501	7420	0.002	0.471	0.007	0.001
Dmean (cGy)	7250	7238	7214	0.253	0.569	0.282	0.105
HI	0.073	0.070	0.070	0.785	0.723	0.802	0.665
Planning Parameters							
Segments	181.00	178.00	126.00	0.003	0.782	0.001	0.001
Mus	972.30	985.20	856.70	0.005	0.689	0.001	0.001
Treamt time(s)	189.6	182.8	426.9	0.001	0.822	0.001	0.001

*The CI of PTVnx included the volume GTVnd and there was no CI for GTVnd.

### HS-VMAT showed significantly lower radiation doses on hippocampus and brain stem when comparing with C-VMAT

**Figure 1:**
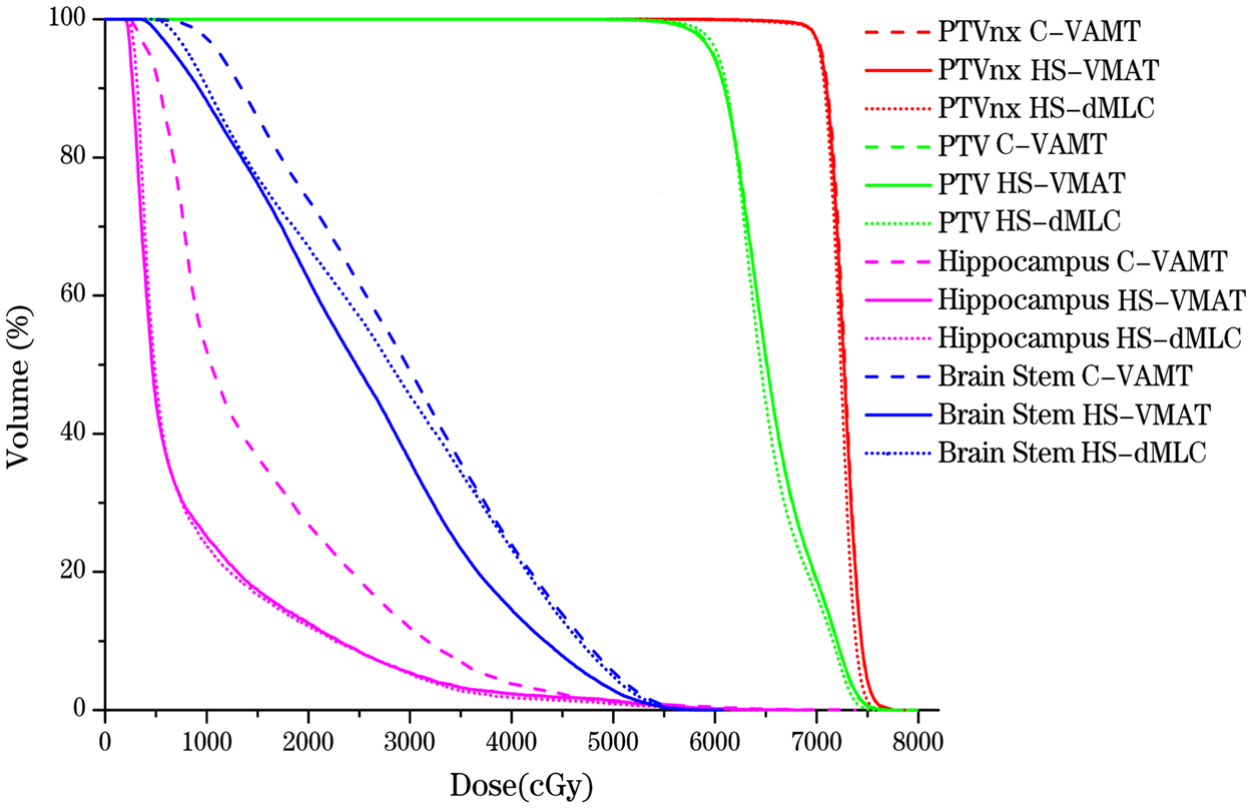
The DVH comparison of three kinds of plans.

**Figure 2:**
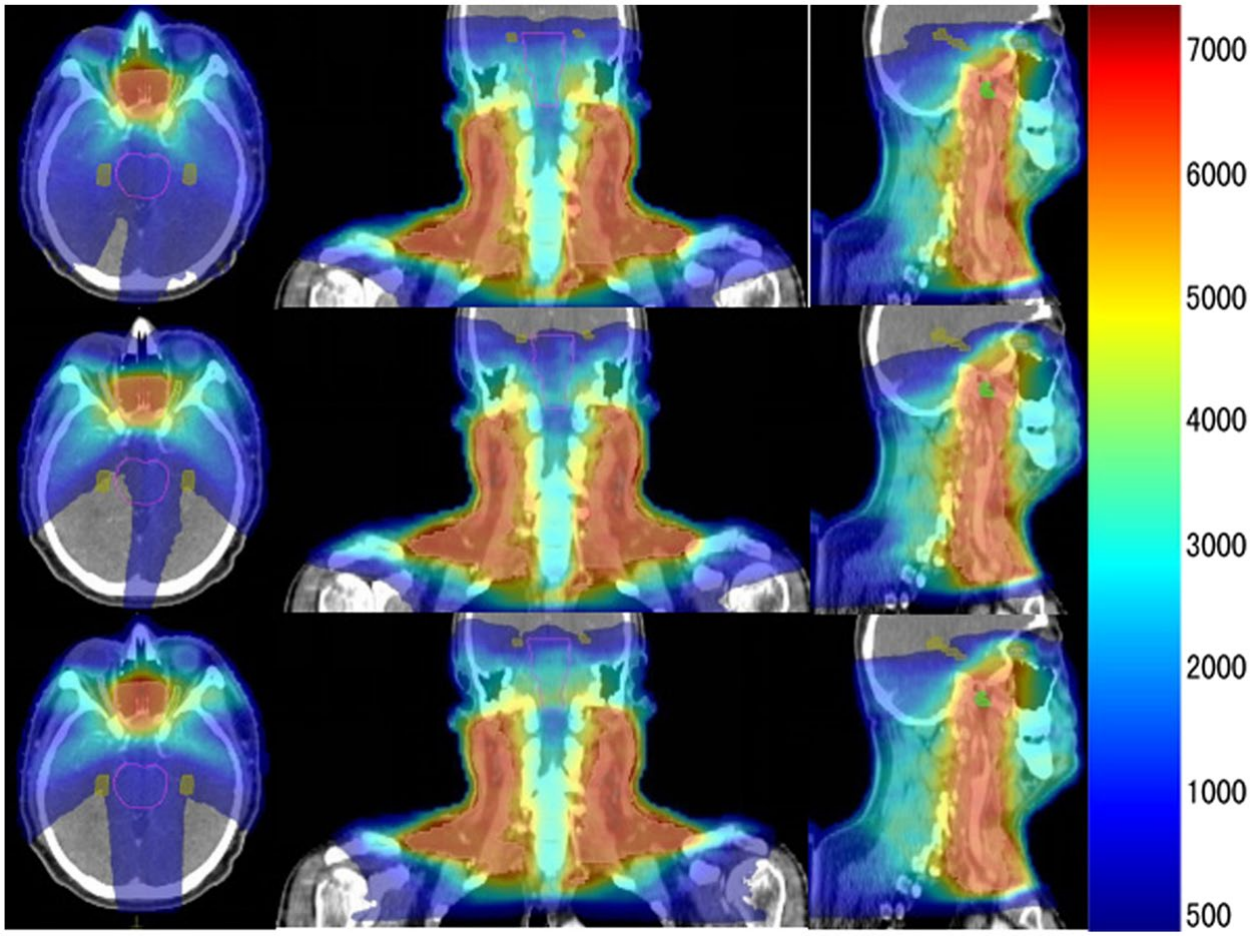
Dose distribution in selected transversal, coronal and sagittal planes for a patient. The first row was from the conventional VMAT plans, the second row from HS-VMAT plans, the third row from HS-dMLC plans.

We then analyzed the radiation doses on hippocampus and brain stem in all the three plans. We found out that C-VMAT presented much higher expected radiation doses on hippocampus as compared to HS-VMAT and HS-dMLC (p < 0.001), with D40%, Dmean, V10, V20 of 1518 cGy, 1379 cGy, 54.1%, 26.2% in C-VMAT, 899 cGy, 642 cGy, 25.1%, 12.6% in HS-VMAT, and 896 cGy, 639 cGy, 23.8%, 12% in HS-dMLC. However, there were no statistical significance in the parameters of V30, V40, V50 in C-VMAT plan when comparing with the other two HS plans. Data were listed in Table 3 and shown in Fig. 1 (Fig. 1 exhibited the DVH comparison of three kinds of plans). What’s more, the dosimetric parameters for brain stem in HS-VMAT plan were also significantly lower in contrast to C-VMAT, indicating a protective role on brain stem in HS-VMAT plan. But in HS-dMLC, only V10 for brain stem showed statistical significance when comparing with C-VMAT (90.3 ± 6.5 vs 97.2 ± 3.42), which implied that HS-dMLC had no advantages in protecting brain stem as compared to HS-VMAT. And such diversity could be seen in the Fig. 2. However, there were no statistically significant differences in the dosimetric parameters of brain stem between HS-VMAT and HS-dMLC (Table 3). Dosimetric parameters for other surrounding OARs, such as cerebellum, parotids, and middle ears, were also analyzed (data in Table 4), and no extreme differences were obtained among all the three plans.

**Table 3:** The doses to brain stem and hippocampus. (Plan A: C-VMAT, Plan B: HS-VMAT, PlanC: HS-dMLC).

	Plan A	Plan B	Plan C	P value			
	mean	mean	mean		A Vs B	B VS C	A Vs C
Brain stem							
V_10_ (%)	97.2	88.1	90.3	0.001	0.001	0.357	0.005
V_20_ (%)	74.2	62.3	67.1	0.032	0.01	0.277	0.109
V_30_ (%)	50.9	35.8	45.5	0.02	0.006	0.066	0.302
V_40_ (%)	24.4	14.4	23.3	0.018	0.01	0.02	0.766
V_50_ (%)	5.7	2.8	4.9	0.061	0.022	0.099	0.484
Hippocampus							
D_mean_ (cGy)	1518	899	896	0.001	0.001	0.978	0.001
D_40%_ (cGy)	1379	642	639	0.001	0.001	0.987	0.001
V_10_ (%)	54.1	25.1	23.8	0.001	0.001	0.796	0.001
V_20_ (%)	26.2	12.6	12.0	0.001	0.001	0.886	0.001
V_30_ (%)	11.4	5.4	5.2	0.127	0.076	0.952	0.068
V_40_ (%)	3.7	2.4	1.8	0.798	0.630	0.848	0.501
V_50_ (%)	1.3	1.4	0.9	0.96	0.935	0.847	0.784

**Table 4:** The doses to other OARs.(Plan A: C-VMAT, Plan B: HS-VMAT, PlanC: HS-dMLC).

	Plan A	Plan B	Plan C	P value			
	mean	mean	mean		A Vs B	B VS C	A Vs C
Cerebellum							
V_10_ (%)	97.3	95.1	96.2	0.451	0.211	0.513	0.542
V_20_ (%)	69.3	57.8	70.2	0.172	0.117	0.094	0.91
V_30_ (%)	27.9	21.7	27.5	0.48	0.279	0.318	0.932
V_40_ (%)	5.5	3.3	5.2	0.343	0.179	0.246	0.849
V_50_ (%)	1	0.5	0.7	0.702	0.404	0.696	0.655
Lens							
D_2%_ (cGy)	715	718	753	0.755	0.782	0.613	0.536
Left parotid							
D_2%_ (cGy)	6533	6530	6601	0.921	0.951	0.564	0.701
D_mean_ (cGy)	3905	3872	4022	0.695	0.936	0.446	0.495
Right parotid							
D_2%_ (cGy)	6532	6508	6004	0.436	0.959	0.278	0.256
D_mean_ (cGy)	4159	3916	4034	0.722	0.424	0.696	0.68
Chaism							
D_2%_ (cGy)	5244	5172	5241	0.788	0.547	0.559	0.985
Optic nevers							
D_2%_ (cGy)	5282	5285	5266	0.938	0.875	0.898	0.901
Middle ears							
D_2%_ (cGy)	6310	6235	6305	0.896	0.713	0.826	0.881
D_mean_ (cGy)	4772	4863	4721	0.823	0.712	0.538	0.795

## Discussion

IMRT (including VMAT) showed obvious advantages in the treatment of irregularly-shaped targets in LA NPC^5–7^, for it had highly conformal dose distribution around the targets and can significantly improve the tumor local control rate and overall survival rate, while reducing the damage to the surrounding OARs. But such radiotherapeutic plans were also reported to have severe side effects, especially on neuro-cognition of memory and social understanding. Later studies^3, 4^ revealed that it was the radiation damage on hippocampus during radiotherapy that caused neuro-cognitive dysfunction.

Hippocampus was not taken into consideration as OARs when designing radiotherapeutic plans in LA NPC patients in either *the experts’ consensus on the organs’ delineation for the nasopharyngeal cancer 2010* ^10^ or the protocol of RTOG 0615^11^. Khodayari *et al*.^15^ assessed the permissible radiation doses for hippocampus in 10 NPC patients, and reported that the mean V20, V40 and Dmean were 72.2%, 22%, 30.27 Gy respectively. Dunlop’ study^16^ proved that significant neuro-cognitive damage would probably occur when the index D40% for bilateral hippocampi increased higher than the threshold of 7.3 Gy (D40% > 7.3 Gy), and they also demonstrated that the higher the radiation dose on hippocampi, the more risky the damage on neuro-cognition. Therefore, if no extra protective measures were taken on hippocampi when designing IMRT plan, actual radiation doses on hippocampus would indeed exceed permissible threshold and cause neuro-cognitive damage of declined memory and social understanding.

Han *et al*.^17^ compared IMRT plans with or without the dose-volume limit to the hippocmpus in 8 LA NPC patients, and showed that the dose-volume limit for the hippocmpus could significantly decrease the radiation dose on the hippocampus, especially in the parameter of the mean V20 which lowered down by 37.25%. Khodayari *et al*.^15^ later found that HS-IMRT could help to greatly diminish the radiation dose on the hippocampus with mean D40% lowering from 23.5 Gy down to 8.6 Gy. Our study here also demonstrated that with the dose-volume limit (D40% was not great than 7.3 Gy) for the hippocampus, the Dmean and D40% of the hippocampus were decreased 41% and 54% in HS-VMAT respectively as compared to C-VMAT. Dosimetric parameters of V10 and V20 for the hippocampus in HS plans also decreased significantly, while V30 and V40 only exhibited a slight decline. Although there were no reports clearly illustrating that reduction of dose parameters such V10 and V20 for hippocampus could help to gain neuro-cognitive protection in LA NPC patients, the mean dose on hippocampi also decreased corresponding to V10 and V20 reduction. Seibert *et al*.^18^ reported that the mean dose on hippocampus positively correlated well with the volume loss on hippocampi, and volume loss have already been defined to protect hippocampus from radiation damage, indicating that the decrease of mean dose can also gain neuro-cognitive protection.

Sudies^15, 17^ before had already illustrated that no matter the IMRT plans spared the hippocampus or not, there was no significant diference on the dose coverage of the targets (PTV, PTVnx, GTVnd), as well as the CI and HI. Our study here also showed that HS plans had no effect on dose coverage of targets in LA NPC patents as compared to C-VMAT, and the dose paremeters of D98%, D50%, D2%, Dmean, CI and HI showed no statistical significance among HS-VMAT, HS-dMLC, and C-VMAT. But there were meaningless differences (like HI of PTV, D2% to the PTVnx) between the plans of HS-VMAT and HS-dMLC, which were caused by different delivery methods.

We also found out that all dosimetric parameters on brain stem (V10~V50) in HS-VMAT were significantly lower than those in the C-VMAT plans. However in HS-dMLC plans, only V10 exhibited a significant decrease to 90.3 ± 6.5% as compared to 97.2 ± 3.42% in C-VMAT plan, and the reason why HS-dMLC failed to exhibit protection on brain stem, we speculated, was that there were only nine fixed beams in HS-dMLC plan. This caused limited directions for irradiation to the targets and it was hard to lower the dose to the hippocampus. Our results were to some extent consistent with what was reported by Dunlop *et al*.^16^, which showed that Dmean for brain stem decreased from 36.1 Gy in conventional plans to 32.3 Gy in HS plans. Yet they failed to exhibit a statistical significance in the dose parameter of V20, which only slightly decreased from 91.8% to 89.5%. However in their study, they included 2 patients with fixed field IMRT and 6 patients with VMAT, which may bias statistical results. Our study also presented similar results of no statistical significance in V20 for brain stem between HS-dMLC and C-VMAT.

Despite the lower doses on hippocampus and brain stem, HS-VMAT showed no difference on other OARs as compared to C-VMAT. Han *et al*.^17^ found that compared to the conventional IMRT plans, the doses on eyes and chaisms decreased in the HS-IMRT plans, but increased on the optic nerves and cochlear. Dunlop *et al*.^16^ reported that HS plans showed decreased radiation dose on cerebellum and brain tissue as compared to conventional plans, but had no influence on eyeball, parotid gland, mandibular. Our study here also showed that no statistical changes were observed in radiation doses on the cerebellum, eyes, optic nerves, chiasm and parotids in all the three plans (Tables 3, 4). Different studies reported quite different dose parameters for targets and surrounding OARs, which may be attributed to different treatment facilities of linear accelerators, therapy planning systems, the prescriptions, the contouring of organs and so on. For example, Dunlop *et al*.^16^ reported that the mean value of V20 for cerebellum was 82.5% in HS plans, and 93.6% in conventional plans. Yet in our study, the mean value of V20 for cerebellum was 69.3% in conventional plans, 57.8% in HS-VMAT, and 70.2% in HS-dMLC respectively. This can explain why radiation doses on cerebellum in our study were much lower than that in Dunlop’.

## Conclusion

In conclusion, the HS plans, whether or not it was VMAT or dMLC, can significantly reduce the radiation doses on hippocampus, but had little influence on the target coverage. HS-VMAT showed more advantage on lowering radiation doses on brain stem as compared to HS-dMLC. Thus, HS-VMAT can help LA NPC patients gain favorable benefits of reducing neuro-cognitive damages.

## Electronic supplementary material

Supplemental Table
