# Supplementary material for: The Hippocampus Sparing Volume Modulated Arc Therapy does not Influence Plan Quality on Locally Advanced Nasopharyngeal Carcinoma Patients
Source: Sci Rep. 2017 Jun 13;7:3443. doi: 10.1038/s41598-017-03517-y (PMC5469746; doi:10.1038/s41598-017-03517-y)
Supplement: Supplementary file 1 — Supplemental Table [file 41598_2017_3517_MOESM1_ESM.doc]

**The Hippocampus Sparing Volume Modulated Arc Therapy does not Influence Plan Quality on Locally Advanced Nasopharyngeal Carcinoma Patients**

Wendong Gu^1*^ Qilin Li^1*^ Dan Xi^1^ Ye Tian^2^ Juncong Mo^1^  Honglei Pei^1^

*1. Department of Radiation Oncology，The Third Affiliated Hospital of Soochow University，The First Peoples' Hospital of Changzhou，Changzhou 213003，China*

*2. Department of Radiation Oncology，The Second Affiliated Hospital of Soochow University，Suzhou 215004，China*

** Two authors contributed equally to this work*

*Corresponding authors: Dr. Pei Honglei，Email: hongleipei@126.com*

Supplemental Table 1：the detailed information for the patients

| Case No. | Age | Sex^a^ | Stage | Volume(cc) | | Invaded structures^c^ |
| --- | --- | --- | --- | --- | --- | --- |
|  |  |  |  | GTV | PTV60 |  |
| Case 1 | 58 | M | 4 | 34.23 | 814.81 | 1,2,3,4,5,6,7 |
| Case 2 | 38 | F | 3 | 21.3 | 884.67 | 1,5,6,8 |
| Case 3 | 53 | M | 3 | 45.48 | 1007.7 | 1,6,7,9 |
| Case 4 | 59 | M | 3 | 51.55 | 882.84 | 1,4,5,8 |
| Case 5 | 43 | M | 4 | 134.54 | 732.54 | 1,2,5,6,7,9,10,11,12,13 |
| Case 6 | 62 | M | 3 | 33.5 | 686.6 | 1,5,6,7 |
| Case 7 | 53 | F | 3 | 27.8 | 710.28 | 1,5,7 |
| Case 8 | 52 | M | 3 | 32.36 | 998.79 | 1,7,9,11 |
| Case 9 | 61 | F | 4 | 71.34 | 860.6 | 1,2,6,7,10,12,13 |
| Case 10 | 67 | M | 4 | 57.3 | 735.67 | 3,5,6,7,11,14 |
| Case 11 | 67 | M | 4 | 63.5 | 536.61 | 2,3,5,6,15 |

a. M: Male, F: Female

b. GTV: gross tumor volume, PTV60: plan target volume (the prescription dose: 60Gy)

c. 1. the base of the skull，2. sphenoid sinus, 3.ethmoid sinus, 4. medial pterygoid muscle,5. longus capitis, 6.parapharyngeal gap, 7. pterygoid process, 8. medial pterygoid plate, 9. oropharynx, 10. the clivus, 11. lateral pterygoid muscle, 12. cavernous sinus, 13. posterior maxillary sinus, 14. pterygopalatine fossa, 15. lateral pterygoid plate
